# Intensive care unit scoring systems outperform emergency department scoring systems for mortality prediction in critically ill patients: a prospective cohort study

**DOI:** 10.1186/2052-0492-2-40

**Published:** 2014-07-01

**Authors:** Erika M Moseson, Hanjing Zhuo, Jeff Chu, John C Stein, Michael A Matthay, Kirsten N Kangelaris, Kathleen D Liu, Carolyn S Calfee

**Affiliations:** 1 Department of Medicine, Division of Pulmonary and Critical Care Medicine, Oregon Health and Science University, 3181 SW Sam Jackson Park Road, UHN67, Portland, OR 97239, USA; 2 Department of Medicine, Division of Pulmonary and Critical Care Medicine, University of California, San Francisco, San Francisco, CA 94143, USA; 3 Department of Anesthesia, University of California, San Francisco, San Francisco, CA 94143, USA; 4 Department of Medicine, Division of Hospital Medicine, University of California, San Francisco, San Francisco, CA 94143, USA; 5 Department of Medicine, Division of Nephrology, University of California, San Francisco, San Francisco, CA 94143, USA; 6 Department of Emergency Medicine, Sutter Medical Center of Santa Rosa, Santa Rosa 95404, USA; 7 Cardiovascular Research Institute, University of California, San Francisco, San Francisco, CA 94143, USA

**Keywords:** APACHE, Emergency medicine, Mortality, Calibration, Intensive care unit, Critical illness, SAPS

## Abstract

**Background:**

Multiple scoring systems have been developed for both the intensive care unit (ICU) and the emergency department (ED) to risk stratify patients and predict mortality. However, it remains unclear whether the additional data needed to compute ICU scores improves mortality prediction for critically ill patients compared to the simpler ED scores.

**Methods:**

We studied a prospective observational cohort of 227 critically ill patients admitted to the ICU directly from the ED at an academic, tertiary care medical center. We compared Acute Physiology and Chronic Health Evaluation (APACHE) II, APACHE III, Simplified Acute Physiology Score (SAPS) II, Modified Early Warning Score (MEWS), Rapid Emergency Medicine Score (REMS), Prince of Wales Emergency Department Score (PEDS), and a pre-hospital critical illness prediction score developed by Seymour et al. (JAMA 2010, 304(7):747–754). The primary endpoint was 60-day mortality. We compared the receiver operating characteristic (ROC) curves of the different scores and their calibration using the Hosmer-Lemeshow goodness-of-fit test and visual assessment.

**Results:**

The ICU scores outperformed the ED scores with higher area under the curve (AUC) values (*p* = 0.01). There were no differences in discrimination among the ED-based scoring systems (AUC 0.698 to 0.742; *p* = 0.45) or among the ICU-based scoring systems (AUC 0.779 to 0.799; *p* = 0.60). With the exception of the Seymour score, the ED-based scoring systems did not discriminate as well as the best-performing ICU-based scoring system, APACHE III (*p* = 0.005 to 0.01 for comparison of ED scores to APACHE III). The Seymour score had a superior AUC to other ED scores and, despite a lower AUC than all the ICU scores, was not significantly different than APACHE III (*p* = 0.09). When data from the first 24 h in the ICU was used to calculate the ED scores, the AUC for the ED scores improved numerically, but this improvement was not statistically significant. All scores had acceptable calibration.

**Conclusions:**

In contrast to prior studies of patients based in the emergency department, ICU scores outperformed ED scores in critically ill patients admitted from the emergency department. This difference in performance seemed to be primarily due to the complexity of the scores rather than the time window from which the data was derived.

## Background

Clinicians and researchers require robust models for mortality prediction in critically ill patients, and multiple scoring systems have been developed for this purpose in both the emergency department (ED) [1-4] and the intensive care unit (ICU) [5-8]. In general, ED-based scoring systems employ a handful of variables that are readily available on all patients, while ICU scoring systems employ a larger number of variables that are frequently available only in those patients that are critically ill (e.g., arterial blood gas measurements). It remains unknown which of these scores perform best in ICU patients.

While it seems intuitive that scores using a larger number of data inputs would perform better than more parsimonious scoring systems, simpler scores may actually outperform more complex scores when the population has been well-defined. For example, the original Acute Physiology and Chronic Health Evaluation (APACHE) score had 34 variables and when reduced to 12 for APACHE II, it performed better in aggregate than did its predecessor [5]. Furthermore, some of the ED scoring systems, though simpler, have been reported to perform as well or better than APACHE II in critically ill emergency department patients [2,9,10]. Greater score complexity increases the barrier to calculation, as it increases the likelihood that some required variables may not be available. If a simpler score using variables available on all patients performed as well as complex ICU scores, it would lower cost and complexity for comparing ICU populations for research purposes. In the realm of benchmarking and quality improvement, comparing populations of ICU patients in terms of their level of acuity and predicted mortality is becoming increasingly common [11,12]. With increased financial pressure on performance measures and hospital benchmarking, there will be strong incentive to find more parsimonious scores that can deliver the same performance as complicated scores in the ICU, and the ED scores may prove a tempting target. Our study seeks to determine whether performing comparisons in ICU patients with simpler scores is legitimate.

We therefore tested the performance of multiple ED and ICU scoring systems in ICU patients admitted directly from the ED, a cohort of patients in which the use of both scores is appropriate. As major new research initiatives such as the renewal of the ARDS network (prevention and treatment of acute lung injury (PETAL)) focus more on the population of critically ill patients admitted via the ED to the ICU, determining the best method to use to adjust for severity of illness in this population is critically important. We hypothesized that the greater complexity of the ICU scores would lead to increased prognostic power compared to the ED-based scoring systems.

## Methods

### Selection of scoring systems

Multiple scoring systems have been developed over the last few decades with the goal of predicting mortality in acutely ill patients. Among the ED-based scores that have been developed, we selected four: the Rapid Emergency Medicine Score (REMS) [1], the Modified Early Warning Score (MEWS) [13], the Prince of Wales Emergency Department Score (PEDS) [2], and a new score focused on variables available to pre-hospital providers developed by Seymour et al. [14]. We compared these ED-based scores with three ICU-based scoring systems: APACHE II [5], APACHE III [8], and the Simplified Acute Physiology Score (SAPS) II [6].

REMS was developed by adding age and peripheral oxygen saturation to the Rapid Acute Physiology Score (RAPS), which was itself derived from APACHE II. We included REMS over RAPS, as REMS recently outperformed RAPS in predicting in-hospital mortality, and the two additional data points are widely available [1]. We used the REMS score based on systolic blood pressure (SBP) used by Goodacre et al. [15] instead of the REMS score based on mean arterial pressure by Olsson et al. [16] as SBP was more frequently available. We included MEWS due to its ease of use and employment of data routinely captured on patients in the ED [17]. The Prince of Wales Emergency Department Score (PEDS) was developed in 2009, and in the single center study in which it was developed, it outperformed APACHE II, REMS, and MEWS in predicting admission to the ICU or death [2]. We also included a newer score developed by Seymour et al. [14] that was developed to predict critical illness in the pre-hospital emergency care of nontrauma patients; in its original publication, this score was also highly predictive of mortality. To our knowledge, neither PEDS nor the Seymour score has been validated by other groups of investigators.

Of the ICU scoring systems, we included SAPS II and APACHE II, as both scores are frequently used in the critical care literature and have performed well in comparison to other critical care scoring systems [18]. Furthermore, APACHE II is frequently the ICU scoring standard to which other scoring systems have been compared [2,10]. We also included APACHE III for further analysis as its performance compared to APACHE II has been mixed. Specifically, APACHE III has been shown to underestimate mortality to a greater extent and has worse calibration than APACHE II in certain disease categories [19-21], but it has better discrimination than APACHE II [18].

A comparison of the different variables employed by the various scores is available in Table 1. Further details on the emergency department scoring systems are included as [see Additional file 1].

**Table 1:** A comparison of the variables included in different scoring systems

**Patient variable**	**REMS**	**MEWS**	**Seymour**	**PEDS**	**SAPS II**	**APACHE II**	**APACHE III**
Temperature	x	x			x	x	x
Respiratory rate	x	x	x			x	x
Mean arterial pressure	x					x	x
Systolic blood pressure		x	x	x	x		
Heart rate	x	x	x		x	x	x
Pulse oximetry (%)	x		x				
Glasgow coma scale total	x		x	x	x	x	x
GCS visual		x					x
GCS motor		x					x
GCS speech		x					x
Age	x		x		x	x	x
Chronic disease and elective postop						x	x
Chronic disease and emerg postop						x	x
Chronic disease and nonoperative						x	x
Metastatic cancer				x	x		x
Hematologic malignancy^a^					x		x
Immunosuppressed						x	x
AIDS					x		x
Hepatic failure or cirrhosis^a^							x
Medical admission					x		
Unscheduled surgery					x		
Serum glucose				x			x
Serum bicarbonate				x	x		
WBC				x	x	x	x
Hematocrit						x	x
Urine output in 24 h					x		x
Serum Cr						x	x
Serum BUN					x		x
Serum potassium					x	x	x
Serum sodium					x	x	x
Serum bilirubin					x		x
PaO2/FiO2					x	x	x
A-a gradient						x	x
pH on ABG						x	x
pCO2 on ABG							x
Acute renal failure						x	x

^a^APACHE III separates ‘Leukemia and multiple myeloma’ from lymphoma and also awards different points to ‘Hepatic failure’ and cirrhosis.

### Data collection

Data were obtained from a prospective observational cohort of critically ill patients admitted to an intensive care unit via the emergency department at an academic, tertiary care medical center (University of California San Francisco, Moffitt-Long Hospital). The hospital has multiple ICUs, and patients are usually cared for by a primary service (such as medicine or cardiology) as well as an intensive care unit team in consultation. All patients admitted to an ICU directly from the ED were consecutively screened for inclusion from October 2008 to July 2011. Patients less than 18 years of age, prisoners, those with a documented pregnancy, trauma patients, and patients with a primary neurologic diagnosis and without other acute medical or surgical complications were excluded. The study was approved by the Institutional Review Board of the University of California San Francisco. All patients or their surrogates provided informed consent for study participation, with the exception of (1) patients who died before they or their surrogate could be approached for informed consent and (2) patients whose critical illness precluded them from providing informed consent and for whom a surrogate could not be identified after 28 days. For these two categories of patients, the IRB approved a waiver of consent.

Clinical data were prospectively collected during hospital admission. Laboratory data were imported directly from hospital clinical software. Severity scores were calculated by computer, and scores were double-checked at random by manual calculation to ensure that manual calculations and computer scores matched. ICU scores were calculated by their respective protocols using the values obtained for the first 24 h in the ICU. We calculated the ED scores from two different time points during the hospital stay. First, the ED scores were calculated from laboratory values and vital signs taken in the ED, as the scores were originally designed. The Seymour score was calculated from triage vitals on arrival to the ED, as it was initially designed for the pre-hospital setting. As a sensitivity analysis, we also calculated the ED scores using the same laboratory data and vital signs used by the ICU scores (that is, data from the first 24 h of the ICU stay) to ensure that any observed differences were due to the scoring system and not the time point of data collected. The Glasgow coma score for both the ED and ICU scores was taken from the exam on admission to the ICU. In calculating APACHE scores, missing data generally resulted in exclusion of that patient from comparison. However, the following values, if missing, were considered normal: albumin, bilirubin, pH, pCO2, and comorbidities. The primary endpoint was 60-day mortality.

### Statistical analysis

Statistical analysis was performed with STATA 12.0 (College Station, TX, USA). Patient characteristics were analyzed using unpaired *t* tests for continuous variables and chi-squared test or Fisher's exact test for categorical variables. ICU scores (APACHE II, APACHE III, SAPS II) and ED scores (REMS, MEWS, PEDS, and Seymour score) were calculated using STATA. Discrimination of the scoring systems was assessed within receiver characteristic (ROC) analyses for each score and corresponding area under the curve (AUC). Specifically, we included each of the individual scores as a single covariate in univariate logistic regression to determine its ability to predict mortality at 60 days. The STATA command ‘roccomp’ was then used to test the equality of the area under the curve for all seven scores.

Calibration of the models was evaluated with calibration plots. In these plots, patients were divided into deciles according to their predicted risk which was then compared with the mean observed mortality at 60 days. Calibration of the model to evaluate the concordance of observed and predicted mortality was further evaluated with the Hosmer-Lemeshow goodness-of-fit test, applying the statistic to a logistic regression using a chi-square with ten groups. Two tailed *p* values less than 0.05 were considered significant.

## Results

A total of 380 patients had mortality outcomes; 118 were missing sufficient data to calculate the ICU scores and were excluded from this analysis, and a further 35 were missing data to calculate ED scores and were therefore similarly excluded. Complete data were available to calculate severity of illness scores on 227 patients. The patient characteristics are shown in Table 2. The majority of patients were admitted to the medical service (67%). The cohort was approximately evenly split between men and women with an average age of 65. Of the patients, 60% had hypertension, 33% had diabetes, 31% had chronic lung disease, 26% had cancer, and 19% had congestive heart failure. Of the 227 patients, 57 (25%) died at 60 days.

**Table 2:** Patient characteristics

**Patient characteristic**	**All**	**Alive at 60 days**	**Dead at 60 days**	***p *** **value**
*N*	227	170	57	
Age	65 ± 17	63 ± 17	70 ± 17	0.01
Gender				
Male	116 (51%)	90 (53%)	26 (46%)	0.52
Female	110 (48%)	79 (46%)	31 (54%)	
Transgender	1 (0.4%)	1 (0.6%)	0 (0%)	
Race				
Caucasian	96 (42%)	71 (42%)	25 (44%)	0.66
African American	34 (15%)	27 (16%)	7 (12%)	
Asian/Pacific Islander	54 (24%)	37 (22%)	17 (30%)	
Hispanic	38 (17%)	31 (18%)	7 (12%)	
Other	5 (2%)	4 (2%)	1 (2%)	
DNR/DNI or comfort measures on admission	44 (19%)	29 (17%)	15 (26%)	0.13
Admitting service				
Medicine	152 (67%)	120 (71%)	32 (56%)	0.14
Surgery	10 (4%)	8 (5%)	2 (4%)	
Cardiology	34 (15%)	21 (12%)	13 (23%)	
Other	31 (14%)	21 (12%)	10 (18%)	
Primary admission diagnosis category				
Respiratory	61 (27%)	44 (26%)	17 (30%)	0.26
Cardiovascular	41 (18%)	29 (17%)	12 (21%)	
ID	47 (21%)	33 (19%)	14 (25%)	
Neurology	26 (11%)	19 (11%)	7 (12%)	
GI	19 (8%)	15 (9%)	4 (7%)	
Other	33 (15%)	30 (18%)	3 (5%)	
Insurance				
Medicaid	41 (19%)	35 (22%)	6 (11%)	0.33
Medicare	86 (41%)	59 (37%)	27 (51%)	
Private insurance	61 (29%)	46 (29%)	15 (28%)	
Other	19 (9%)	15 (9%)	4 (8%)	
None	4 (2%)	3 (2%)	1 (2%)	
Coronary artery disease	41 (18%)	32 (19%)	9 (16%)	0.61
Congestive heart failure	43 (19%)	29 (17%)	14 (25%)	0.21
Hypertension	136 (60%)	103 (61%)	33 (58%)	0.72
Chronic lung disease	71 (31%)	52 (31%)	19 (33%)	0.70
Chronic liver disease	14 (6%)	12 (7%)	2 (4%)	0.53
Diabetes	75 (33%)	57 (34%)	18 (32%)	0.79
Malignancy	59 (26%)	39 (23%)	20 (35%)	0.07
Immunosuppressed	36 (16%)	26 (15%)	10 (18%)	0.69
ESRD	20 (9%)	14 (8%)	6 (11%)	0.56

The above categories represent patient comorbidities on admission.

**Figure 1:**
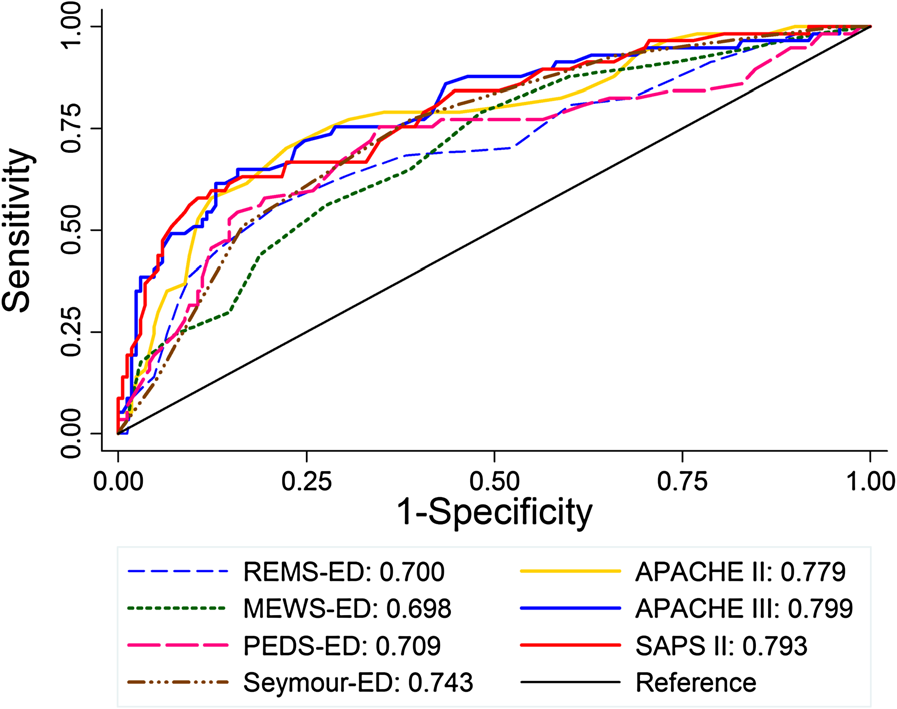
Receiver operating characteristic curves for ICU-based and ED-based scoring systems.

When all seven scores were compared using ROC curves, there were significant differences in discrimination among them (*p* = 0.01; Figure 1). This difference in discrimination appeared to be driven by differences between the ED-based scores and the ICU-based scores. Specifically, when compared to the best performing ICU-based scoring system, APACHE III, which had an area under the ROC curve (AUC) of 0.799, the ED-based scores REMS, MEWS, and PEDS had significantly lower AUCs (AUC 0.698 to 0.709; *p* = 0.005 to 0.01 for comparison of ED scores to APACHE III). The Seymour score (AUC 0.743) had the highest AUC of the ED scores. Though not significantly better than the other ED scores (*p* = 0.45), it was also not significantly different from APACHE III (*p* = 0.09). There were no differences in discrimination among the ED-based scoring systems including Seymour (*p* = 0.45) or among the ICU-based scoring systems (AUC 0.779 to 0.799; *p* = 0.60). The results of the main analysis did not differ when in-hospital mortality instead of 60-day mortality was used as the end point (data not shown).

In order to determine whether these differences in discrimination were driven by the scoring systems themselves or by the time period from which the data inputs were derived, we next used data from the first 24 h of the patient's ICU stay to calculate the ED-based scores (instead of data from the ED itself, as the scores were originally designed). Calculated with ICU data, the AUCs for all the ED scoring systems improved slightly, by 0.004 to 0.038 (Table 3), but this improvement was not statistically significant (*p* = 0.21 to 0.87). There were still significant differences among the seven scores (*p* = 0.04); however, REMS and the Seymour score were not significantly different from APACHE III when calculated with ICU data (*p* = 0.07 and 0.32, respectively).

**Table 3:** Area under the curve (AUC) of ED and ICU scoring systems

**AUC (95% CI)**	**All population (*N*** **= 227)**		**MICU population (*N*** **= 152)**	
**Score**	**ED data**	**ICU data**	**ED data**	**ICU data**
REMS	0.700	0.738	0.740	0.777
	(0.617, 0.782)	(0.662, 0.813)	(0.640, 0.840)	(0.687, 0.867)
MEWS	0.698	0.729	0.733	0.764
	(0.621, 0.776)	(0.652, 0.806)	(0.631, 0.835)	(0.672, 0.857)
PEDS	0.709	0.712	0.744	0.730
	(0.623 – 0.794)	(0.632, 0.793)	(0.633, 0.854)	(0.624, 0.836)
Seymour	0.743	0.767	0.753	0.758
	(0.674, 0.813)	(0.704, 0.829)	(0.666, 0.841)	(0.678, 0.838)
APACHE II	-	0.779	-	0.833
		(0.707, 0.851)		(0.757, 0.909)
APACHE III	-	0.799	-	0.841
		(0.728, 0.870)		(0.766, 0.915)
SAPS II	-	0.793	-	0.830
		(0.722, 0.863)		(0.751, 0.909)

Confidence intervals are in parentheses. *REMS* Rapid Emergency Medicine Score, *MEWS* Modified Early Warning Score, *PEDS* Prince of Wales Emergency Department Score, *APACHE* Acute Physiology and Chronic Health Evaluation, *SAPS* Simplified Acute Physiology Score.

As a sensitivity analysis, we tested the discrimination of the scoring systems in patients admitted to the medical ICU (MICU) (*n* = 152). While most of the scores had slight increases in the AUCs in this population, similar trends in performance were seen as in the broader population (Table 3). Specifically, all the ED-based scores had significantly lower AUCs than APACHE III (*p* = 0.02 to 0.046). Of note, in this smaller sample, the *p* value for the overall comparison of the seven scores was 0.07. When calculating scores using ICU data in the MICU population (Table 3), the performance of REMS and MEWS improved (AUCs 0.777 and 0.764, respectively) and no longer reached criteria for significance in their difference from APACHE III (*p* values 0.10 and 0.07). The performance of the Seymour score (AUC 0.758) was worse than in the general ICU population and different from APACHE III in this subgroup (*p* = 0.05). PEDS also remained significantly different with an AUC of 0.730 (*p* = 0.005).

**Figure 2:**
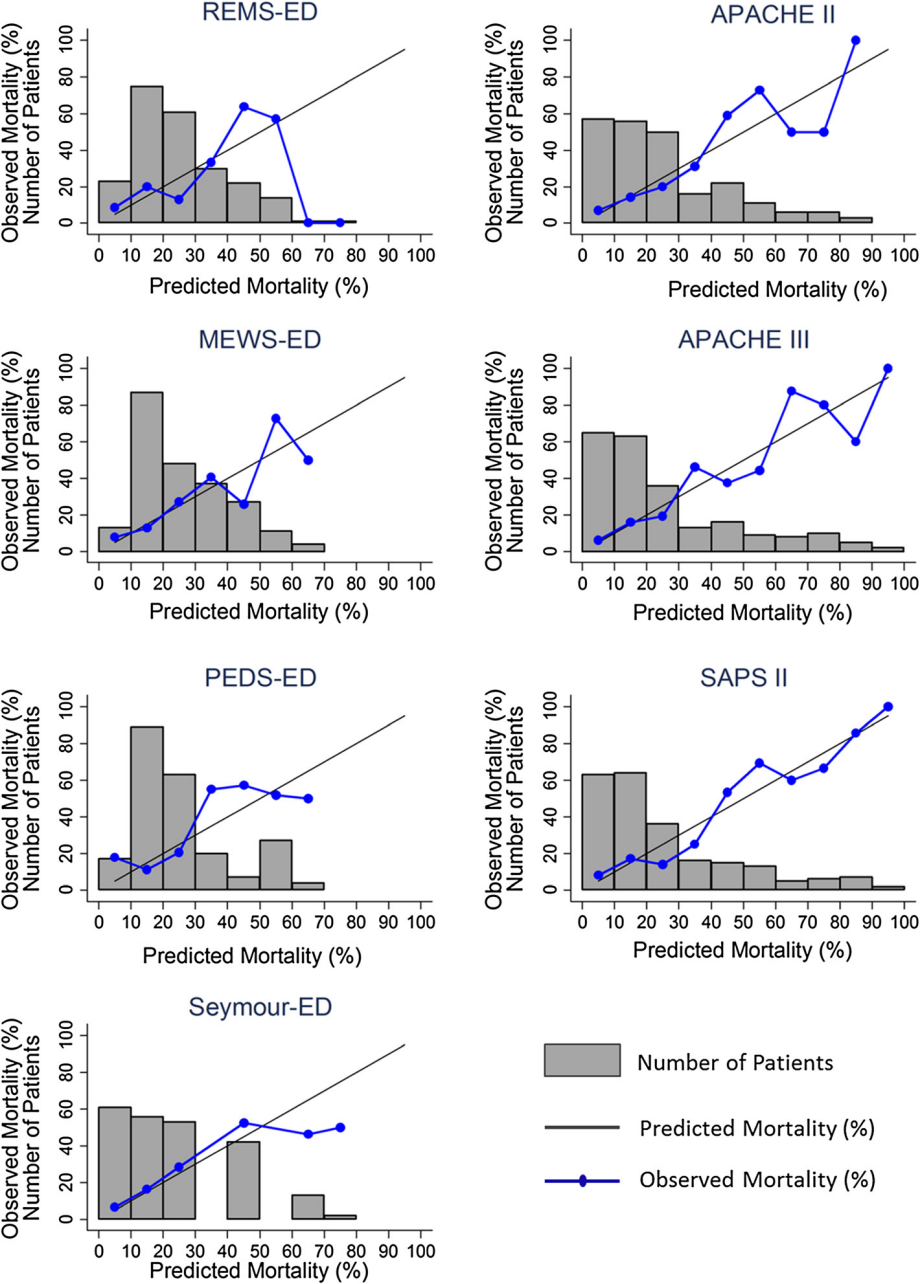
Calibration: observed mortality vs. expected mortality.

We next evaluated the calibration of the models using both the Hosmer-Lemeshow goodness-of-fit test and a visual inspection of mortality by deciles of each score. All of the scores passed the Hosmer-Lemeshow goodness-of-fit test, with *p* values > 0.05 (0.06 to 0.86) in the general and MICU population although several scores had borderline *p* values (Table 4). PEDS was the lone exception; when calculated in MICU patients, it had a *p* value of 0.048. On visual inspection of the deciles, the ICU scores in general appeared to have a more appropriate slope with observed mortality more closely following predicted mortality (Figure 2).

**Table 4:** Calibration of scoring systems evaluated by Hosmer-Lemeshow goodness of fit

**Score**	**All population (*N*** **= 227)**		**MICU population (*N*** **= 152)**	
	**ED data**	**ICU data**	**ED data**	**ICU data**
**REMS**	0.28	0.54	0.33	0.77
**MEWS**	0.53	0.60	0.68	0.26
**PEDS**	0.18	0.83	0.048	0.57
**Seymour**	0.60	0.06	0.58	0.15
**APACHE II**	-	0.48	-	0.86
**APACHE III**	-	0.06	-	0.88
**SAPS II**	-	0.16	-	0.84

*REMS* Rapid Emergency Medicine Score, *MEWS* Modified Early Warning Score, *PEDS* Prince of Wales Emergency Department Score, *APACHE* Acute Physiology and Chronic Health Evaluation, *SAPS* Simplified Acute Physiology Score.

## Discussion

Despite the previous studies demonstrating the equivalence of ED and ICU-based scoring systems for patients in the emergency department [2,10], we found that ICU scoring systems outperform ED scoring systems in critically ill patients admitted from the ED. The finding that the ICU scores had superior performance despite the time at which they were calculated, whether with initial data or at 24 h, suggests that it is the complexity of these scores, not the time at which they are calculated, that provides their prognostic power. To our knowledge, this report is the first to compare multiple ED and ICU scoring systems in a cohort of ICU patients admitted only through the ED, as well as the first external validation of the PEDS and Seymour scores. These findings have important implications for estimating prognosis for a patient early in their admission to the hospital, as this comparison evaluates patients who have presented in a critically ill fashion. It does not include those who are transferred from another hospital or from the general wards, in which some of the clinical disorder may have evolved in the hospital setting. Particularly at an academic referral center, the impact of including critically ill patients transferred from another hospital can affect outcomes for a given population [22].

Scoring systems will continue to be applied widely to compare different patient populations for research and increasingly for benchmarking and quality metrics, and this study illustrates many of the important statistical and methodological concerns that must be addressed when employing different scoring systems. The accuracy of predictive models or prognostic scoring systems should ideally include assessment of both discrimination and calibration [23]. Discrimination examines how well the score can separate those patients who do and do not have the outcome of interest—in this case, death. In contrast, calibration is a measure of how well the probability of the outcome (death) predicted by the score agrees with the actual observed risk. The Hosmer-Lemeshow test, a standard measure of calibration, compares the predicted mortality with the actual mortality in each decile of the sample. Both discrimination and calibration are important to determine whether a score is appropriate to use in a given population. For quality assurance and performance comparisons, calibration may be more important than discrimination, as it has been found to be more sensitive to differences in hospital mortality [24]. In fact, Castella et al. compared the performance of multiple ICU scoring systems and concluded that the models should be well-calibrated to the population at hand before their discrimination can be meaningfully assessed [25]. By the Hosmer-Lemeshow test, all scoring systems showed acceptable calibration in our general population and subsets (with the exception of PEDS with a *p* value of 0.048 in MICU patients). At the same time, with a relatively modest sample size, the power of the Hosmer-Lemeshow test to detect model miscalibration is somewhat limited [26], so we also provide figures to allow visual inspection of the calibration data. This visual inspection (shown in Figure 2) suggests that the ICU scores may be better calibrated to critically ill patients than the ED scores, particularly at the upper score range in the sickest patients with greater disease severity. It may be that the additional complexity of the ICU scores and the additional clinical information account for the better mortality prediction. However, it should be noted that no score is perfectly calibrated, and these scoring systems are better used on the population level than at the level of the individual patient.

These results also illustrate the importance of clearly defining a patient population before applying scoring systems. For example, APACHE II may not perform as well in certain patient subgroups, including some postoperative patients [27], trauma patients [28], and certain subsets of neurosurgical patients [29]. Our patient population excluded trauma patients and patients with isolated neurological problems, which may explain the excellent performance of APACHE in our cohort. Interestingly, the AUC for the scoring systems generally improved slightly when calculated in MICU patients, particularly when calculated with ICU data. In this cohort, cardiology patients were the largest group excluded when narrowed to the MICU population. The improved performance of REMS in the MICU subgroup is therefore not surprising since REMS was derived from APACHE II, which has a varied performance in critically ill cardiology populations [30-32]. REMS and MEWS were also derived from populations of medical or nonsurgical admissions, which likely explained their improved performance when the population was narrowed to MICU patients alone. This finding is also consistent with previous reports that REMS has good discrimination and calibration in predicting mortality in septic patients admitted to the general medicine wards [33].

This study also draws attention to the importance of validating scoring system results in an outside cohort. PEDS in particular seemed to be a promising score, outperforming REMS, MEWS, and APACHE in its derivation cohort [2]. However, its performance in our cohort was decidedly worse. This decline in score performance in a replication sample has been reported in other scoring systems, with the American-derived APACHE scoring systems performing differently in UK populations [18] and in the East [34] and Southeast Asia [35]. The Seymour score appeared to have an intermediate performance between the older ED scores and the ICU scoring systems. Unlike the other ED scores, it did not differ significantly in terms of discrimination from the ICU scoring systems. It may be that its improved performance in our study was due to the fact that the cohort from which it was originally derived and validated may be more similar to our own cohort, as it was more recently derived than the other ED scores and is also located on the West Coast of the United States.

Our study has some limitations. It is a single center study at an academic medical center; therefore, additional replication in separate cohorts from other centers should be carried out. Furthermore, the relatively modest sample size may have precluded detection of small differences in discrimination between scores. It is also important to note that a large sample size may also make calibration differences more significant; as has been noted previously, the calibration of these scores might decline in larger cohorts [24]. Furthermore, Glasgow coma score (GCS) data were only available from admission to the ICU; it may be that the ED scores would perform better if the ED GCS data were available.

Finally, we should note that ED scoring systems have been used for multiple purposes, including prediction of long-term outcomes as well as need for critical illness [13,14]. While ED scores did not perform as well as ICU scores for mortality prediction in this population, they may have additional value for mortality prediction in other hospitalized patients, such as patients triaged by rapid response, though these populations are beyond the scope of this manuscript.

## Conclusions

In contrast to prior studies of patients based in the emergency department, ICU scoring systems outperformed ED severity scores in critically ill patients admitted from the emergency department. This difference in performance appears primarily due to the complexity of the scores rather than the time window from which the data were derived. Among the more parsimonious scoring systems, the Seymour score shows promise in mortality prediction for critically ill patients, while in MICU patients, simpler scores like REMS may perform reasonably well. These results emphasize the importance of repeated validation of prognostic scoring systems as well as the major differences in scoring system performance that can result from application to different patient populations.

## Abbreviations

APACHE: Acute Physiology and Chronic Health Evaluation; AUC: Area under the curve; ED: Emergency department; ICU: Intensive care unit; MEWS: Modified Early Warning Score; PEDS: Prince of Wales Emergency Department Score; REMS: Rapid Emergency Medicine Score; ROC: Receiver operating characteristic; SAPS: Simplified Acute Physiology Score.

## Competing interests

The authors declare that they have no competing interests.

## Authors’ contributions

EM participated in the design of the study and drafted the manuscript. CC participated in the design of the study, the design and recruitment of patients for the patient cohort, and the design of the statistical analyses and heavily reviewed and revised the manuscript. HZ performed the statistical analysis. JC, KK, KL, MM, and JS participated in the design of the study and reviewed and revised the manuscript. All authors read and approved the final manuscript.

## Supplementary Material

Additional file 1 Emergency department scoring system details—this file shows how individual ED scoring systems are calculated. Click here for file
