# Supplementary material for: Intensive care unit scoring systems outperform emergency department scoring systems for mortality prediction in critically ill patients: a prospective cohort study
Source: J Intensive Care. 2014 Jul 1;2:40. doi: 10.1186/2052-0492-2-40 (PMC4424730; doi:10.1186/2052-0492-2-40)
Supplement: Additional file 1 — Emergency department scoring system details—this file shows how individual ED scoring systems are calculated. [file 2052-0492-2-40-S1.pdf]

Appendices- Individual ED based scoring systems

Appendix 1: REMS Scoring System

Physiological variable	High abnormal range					Low abnormal range			.
	**+4**	**+3**	**+2**	**+1**	**0**	**+1**	**+2**	**+3**	**+4**
Body temperature	> 40.9	39–40.9		38.5–38.9	36–38.4	34–35.9	32–33.9	30–31.9	<30
Mean arterial pressure	>159	30–159	110–129		70–109		50–69		<49
Heart rate	>179	140–179	110–139		70–109		55–69	40–54	<39
Respiratory rate	> 49	35–49		25–34	12–24	10–11	6–9		<5
Peripheral O2 sat	<75	75–85		86–89	>89				
Glasgow coma score	<5	5–7	8–10	11–13	>13				

Points for age have been assigned as follows (age, points): <45 ->0; 45–54->2; 55–64->3; 66–74->5; >74->6.

Appendix 2: PEDS Scoring System

Variable	Value	Derived Score
SBP	> 140	-2
	<100	3
GCS	9-12	4
	<8	23
Glc (mmol/l)	>7	4
	<3.2	6
HCO3 (mmol/l)	>26	7
	<22	16
WBC(x10^9/l)	>10.7	3
	<4	6
Metastatic Cancer	Present	4

Appendix 3: MEWS Scoring System

Physiologic variable	Points:	3	2	1	0	1	2	3
Systolic Blood Pressure		<70	71-80	81-100	101-199		≥200	
Heart Rate			<40	41-50	51-100	101-110	111-129	≥130
Respiratory Rate		<9			9-14	15-20	21-29	≥30
Temperature (deg C)		<35			35-38.4		≥38.5	
AVPU Score					**A**lert	Reacts to **V**oice	Reacts to **P**ain	**U**nresponsive

Appendix 4: Seymour Scoring System

Variable	0	1	2 .
Age	< 45	≥45	
RR	12-23	<12 or 24-36	>36
SBP	≥91	≤90	
HR	<120	≥120	
Peripheral O2 sat	≥ 88	<88	
Glasgow coma score	15	8-14	<8
